# In-tube dynamic extraction for analysis of volatile organic compounds in honey samples

**DOI:** 10.1016/j.fochx.2022.100337

**Published:** 2022-05-18

**Authors:** Wiebke Kaziur-Cegla, Maik A. Jochmann, Karl Molt, Andreas Bruchmann, Torsten C. Schmidt

**Affiliations:** a Instrumental Analytical Chemistry, University of Duisburg-Essen, Universitätsstrasse 5, Essen D-45141, Germany; b Centre for Water and Environmental Research (ZWU), University of Duisburg-Essen, Universitätsstr.2, Essen 45141, Germany; c IWW Zentrum Wasser, Moritzstr. 26, Mülheim an der Ruhr 45476, Germany; d Axel Semrau GmbH&Co.KG, Stefansbecke 42, Sprockhövel 45549, Germany

**Keywords:** In-tube extraction, ITEX-DHS, Solvent-less microextraction, GC–MS, Honey analysis, Linear discriminant analysis

## Abstract

Honey is the oldest and nowadays widely used natural sweetener for food worldwide. Its composition is associated with its botanical and geographical origin and honey is often mislabeled and has a high potential for food fraud. Thus, quick easy and sensitive analyses are required. For the first time, we developed and applied an automated, fast, sensitive and robust, in-tube extraction dynamic headspace in-tube extraction-dynamic headspace (ITEX-DHS) method for a variety of Honey containing VOCs in connection with GC–MS. Another advantage of ITEX is, that it is a green analytical solventless method. The method provides very low method detection limits (MDL) from 0.8 to 47 ng g^−1^ for VOCs in honey samples as well as very good repeatabilities with averages below 9 % RSD. Recoveries are between 83 and 100 %. Only octanal possess a repeatability 13 % and a recovery of 62 % due to its high polarity. 38 honey samples were measured after method validation. Four acacia honeys (A), six forest honeys (F) and 22 blossom honeys (B). The type of six honeys was not known (U) but could be predicted with the help of a linear discriminant analysis (LDA). The LDA was carried out with the three groups (A, B, F) leading to a proportion of correct predictions of 90.6 %. With the help of a scatterplot, two of the unknown samples were classified as forest honeys and four of them as blossom honeys.

## Highlights

• In-tube dynamic extraction was applied for volatile compounds of honey.
• ITEX-DHS is a sensitive and robust alternative to other headspace methods.
• 38 supermarket available honey samples where measured.
• Linear discriminant analysis was applied to find similarities.

## Graphical abstract

**Figure:**
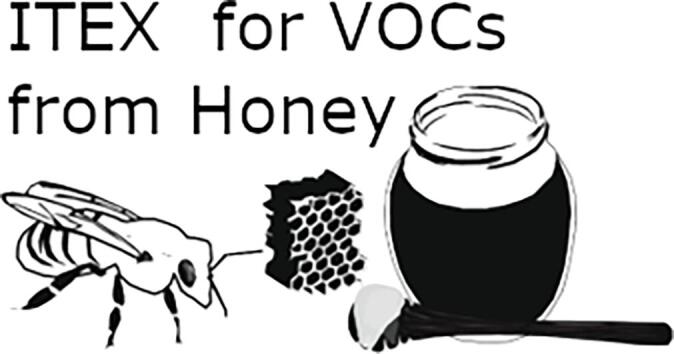

## 1 Introduction

Honey is a natural product which can be used by humans without any processing (Iglesias & de Lorenzo, 2004). Regarding to the Commission of the European Union honey consists essentially of different sugars as well as other substances such as organic acids, enzymes and solid particles derived from honey collection. The color of honey varies from nearly colorless to dark brown as well as consistency can be fluid, viscous or partly to entirely crystallized. Honey shall not have any food additives and it should be prevented from heat, as heat destroys or inactivates the natural enzymes present in the honey (European Union, 2002). Flavor and aroma derive from the plant’s origin, just as soil and climate can cause melliferous flora (Iglesias et al., 2004, Union, 2001). Honey can be distinguished into two groups: monofloral honey and honey blends. The latter are produced in areas where no flower predominates (Babarinde, Babarinde, Adegbola, & Ajayeoba, 2011). Thus the composition of honey is tightly associated to its botanical and geographical origin, as some honey components come from the plants, some from the honeybees and some are due to the biochemical reactions which take place during honey maturation (Anklam, 1998, Iglesias et al., 2004).

The traditional analysis of the floral and geographical origin from honey is done by melissopalynology, which is the analysis of the pollen present in honey using a microscopic examination. The main drawbacks of this technique are that it is very time consuming and requires very experienced analysts. Furthermore, it is dependent on the expert’s interpretation (Ohe, Oddo, Piana, Morlot, & Martin, 2004).

Another approach to identify the honey’s origin is to analyze the volatile organic compounds (VOCs) present in the honey (Baroni, Nores, Díaz, Chiabrando, Fassano, Costa, & Wunderlin, 2006). VOCs, like aldehydes, hydrocarbons, alcohols, ketones, acids and esters, can derive from the plant or nectar source, from the transformation processes of plant compounds, from heating or from microbiological or environmental contamination (Barra et al., 2010, Jerkovic and Marijanovic, 2009). The aroma depends on the different VOCs present in the honey, as well as they influence the taste and flavor. As there are not many marker compounds known for the different monofloral honey types, honey is one of the most frequently adulterated food products. Most often the botanical source is declared as monofloral, when mixed with cheaper honey blends. Therefore, several studies have been carried out to identify marker components for specific monofloral honeys (Alissandrakis et al., 2007, Babarinde et al., 2011, Guyot et al., 1999, Jerković et al., 2006, ODEH et al., 2007). Amino acids play an important role in the ripening of honey, which enter the honey through the saliva and gastric juice of the honey bees and react with reducing sugar species according to the Maillard reaction (da Silva, Gauche, Gonzaga, Costa, & Fett, 2016).The antibacterial and antioxidative activity of honey play a major role in food preservation and human health and is due to polyphenols like flavonoids, phenolic acids and phenolic acid derivates (Lamien-Meda et al., 2005, Tomás-Barberán et al., 2001).

As volatile compounds play an important role in assessing the origin of honey (Radovic, Careri, Mangia, Musci, Gerboles, & Anklam, 2001), microextraction techniques like headspace solid phase microextraction (HS-SPME) can be used prior gas chromatography coupled to a mass spectrometer (GC–MS). One of the main advantages of headspace analysis is that it can be carried out on untreated samples (Piasenzotto, Gracco, & Conte, 2003).

In-tube extraction (ITEX) is a dynamic headspace (DHS) approach, which is fully automated for PAL-type autosamplers and uses a sorbent filled trap with a fixed steel needle attached to a gastight syringe, which can be heated for thermal desorption. Enrichment of analytes takes place by repeated pumping of the sample’s headspace trough the sorbent trap by aspirating and dispensing of the syringe. The injection takes place in the GC injection port by heating the trap to the set desorption temperature and aspiration of a gas, either a portion of the sample’s headspace or a carrier gas. Afterwards the trap is cleaned by heating it out whilst flushing it with nitrogen through the syringe side-port hole (Jochmann et al., 2008, Laaks et al., 2010). ITEX-DHS is a new, fully automated and green solvent free analytical method. Compared to more fragile SPME fibers, SPME needles possess a longer lifetime. So far ITEX-DHS was not applied for the analysis of the VOC fraction in honey samples.

The aim of this study was to show the applicability for the analysis of VOCs in honey with a dynamic headspace approach like ITEX-DHS. Therefor, 38 honey samples were analyzed with ITEX-DHS GC–MS. Additionally as application, a linear discriminant analysis (LDA) was conducted, to show a way of grouping the different honey types and to identify unknown honey samples.

## 2 Materials and methods

### 2.1 Chemicals and reagents

Methanol (99.8%) from Fisher Scientific (Loughborough, UK) was used to prepare stock solutions. Milli–Q water was used from a water purification system (Purelab ultra, Elga, High Wycombe, UK). 2–Phenylethanol (99%), linalool oxide (97%), benzoic acid (99.5%), carvacrol (98%), octanoic acid (96%), ethanol (98%), octane (99%), octanal (99%), benzaldehyde (99 %), dimethylsulfide (98 %), nonanol (98%) and nonanoic acid (99.5 %) were purchased from Sigma-Aldrich (Steinheim, Germany), thymol (99%) from Honeywell Riedel-de Haën AG (Seelze, Germany), phenylacetic acid (99 %) from Alfa Aesar (Karlsruhe, Germany) and 2-Butanol from Fluka (Darmstadt, Germany). Benzaldehyde-d_6_ (99%) from Sigma-Aldrich was used as internal standard. Compound related CAS numbers, boiling points logarithmic air − water (log *K_aw_*) partitioning constants, Satchenow (salting out with NaCl, K_s_) constants and used quantifier and qualifier ions can be found in Table S1. Log *K_aw_* and *K_s_* were calculated using PP-LFERs database (Ulrich, Endo, Brown, Watanabe, Bronner, Abraham, & Goss, 2017). Sodium chloride (>99.5 %, NaCl) purchased from Bernd Kraft (Duisburg, Germany) was used to enhance the ionic strength of the honey samples. Therefore, a 25% (w/v) NaCl solution was prepared weekly by dissolving 125 g of NaCl in 500 mL Milli-Q water.

### 2.2 Stock solutions and standard mixtures

Stock solutions of all 15 analytes were prepared by weighing 10 mg of the pure substance in a 10 mL volumetric flask and diluting it with methanol to a final concentration of 1 gL^−1^ in 10 mL. A standard mix of the 15 analytes was prepared with a concentration of 50 mg/L by transferring 250 µL of each analytes stock solution into a 5 mL volumetric flask and diluting it with methanol. The standard mix was prepared monthly. All solutions were stored in 20 mL amber glass vials sealed with magnetic screw caps with butyl rubber/PTFE septa (BGB Analytik, Rheinfelden, Germany) at 4 °C in the refrigerator. Lower concentrations for method validation were prepared likewise by dilution with 25 % (w/v) NaCl to the required concentration levels.

### 2.3 Honey samples

38 honeys were purchased from supermarkets and local beekeepers. Five different types of honey were covered with the selection: Acacia honey (4), blossom honey (22), forest honey (6). Additionally, there were six honeys, where the type was not defined (U). The origin from most of the honeys was defined as a mixture of honey from EU-countries and non-EU countries. One blossom honey was from Tostedt, Germany, one from Bausendorf, Germany, one from Essen, Germany, one from Niedersachsen and Schleswig-Holstein, Germany, and one from Asiago, Italy. One forest honey was produced in Asiago, Italy and one in Westerkappeln, Germany and honey from Essen, Germany. In Table S1 in the supporting information a complete list of the included honeys is shown.

Honey samples were prepared by weighing 1 g honey into 20 mL amber screw cap glass vials (BGB Analytik GmbH (Böckten, Switzerland). PTFE laminated 8×3 mm magnetic stir bars (VWR International GmbH, Darmstadt, Germany), as well as 10 mL of 25% (w/v) NaCl solution were added to each vial. The vials were closed by magnetic screw caps with rubber/PTFE septa (BGBAnalytik AG, Boeckten, Switzerland) and placed onto the autosampler tray for ITEX-DHS. For quantification.

### 2.4 ITEX-Dynamic headspace

For extraction of the VOCs from the headspace a Tenax TA ITEX trap (BGB Analytik, Adliswil, Switzerland) and a 1.3 mL Headspace syringe (CTC Analytics, Zwingen, Switzerland) were used. The Tenax TA material (polydiphenylene oxide) possess a particle size of 80/100 mesh with a surface area of 35 m^2^g^−1^ and a bulk density of kgm^−3^. The maximum operation temperature is 350 °C.

The samples were incubated at 70 °C for 30 min while stirring at 500 rpm. Meanwhile the ITEX trap was cleaned for 10 min with a trap temperature of 300 °C. After incubation, the ITEX needle was injected into the headspace of the vial with syringe and trap temperatures of 70 °C. Extraction was performed with 65 extraction cycles with an extraction volume of 1 mL and a speed of 100 µL/s. Desorption took place in the GC-injection port with a trap temperature of 300 °C and a flow of 100 µL/s.

### 2.5 Instrumentation

All samples were measured using a Trace GC Ultra (S + H Analytik, Mönchengladbach, Germany) coupled to a single quadrupole mass spectrometer (DSQ II, S + H Analytik). The GC was equipped with a PAL Combi-xt autosampler (Axel Semrau, Sprockhövel, Germany), a split/splitless injector (SLL), and an Optic 3 injector (Axel Semrau) with a cryofocussing unit. The ITEX2 option for the autosampler consisted of a heatable syringe holder, a 1.3 mL syringe with a side port, and a trap heater (CTC Analytics). Additionally, a single magnet mixer (SMM) was attached to the autosampler. Data acquisition and processing were carried out using XCalibur Data System (Version 2.2, ThermoFisher Scientific), ITEX2 procedures were controlled with PAL cycle composer (CTC Analytics) and the temperature for the Optic 3 was controlled with ATAS evolution workstation. For the separation of the analytes an Optima FFAPplus fused-silica capillary column from Macherey-Nagel (60 m × 0.32 mm I.D., 0.5 µm film thickness, Macherey-Nagel, Düren, Germany) was used.

**Fig. 1:**
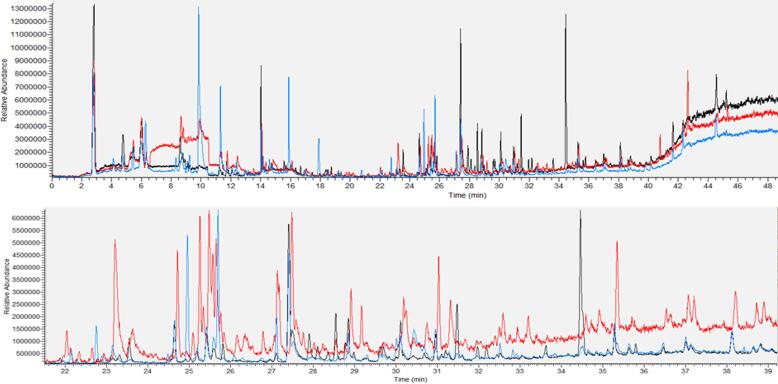
Overlaid chromatograms of acacia honey (orange), blossom honey (black) and forest honey (blue). The chromatogram at the top shows the complete run, the one at the bottom is zoomed in to show the high number of detectable peaks.

The injector was used in splitless mode at a temperature of 300 °C and was equipped with a 2.0 mm I.D. deactivated splitless liner (Restek, Bellefonte, USA). The cryotrap temperature was set to –20 °C with a hold time of 2 min. After the transfer time, the split was opened at 50 mL/min and the cryotrap was heated to 250 °C with a rate of 50 °C/s. Constant column flow of 1.5 mL He 5.0 (Air Liquide, Oberhausen, Germany) was set. The initial GC oven temperature was set to 35 °C and hold for 5 min. Then the temperature was raised to 110 °C with a ramp of 7 °C/min and hold for 2 min. Afterwards temperature was raised to 200 °C with 5 °C/min and hold for 4 min, before being raised with a ramp of 10 °C to the final temperature of 230 °C which was hold for further 2 min, resulting in a total GC run time of nearly 45 min. In Fig. 1 a full scan chromatogram a combined chromatogram of acacia honey, blossom honey and forest honey.

The temperatures for the transfer line and the ion source were set to 260 °C and 230 °C, respectively. The MS was operated in electron impact ionization mode (EI) at 70 eV. Full-scan mode (*m*/*z* = 40–200, 500 amu/s) was used for all measurements. The scan rate was 500.0 amu/s by 2.7473 scans/s. For the quantification SIM Mode was used. The quantifier and qualifier masses are tabulated in Table S1.

## 3 Results and discussion

### 3.1 Method validation

For the validation of the method, the method detection limit (MDL), the repeatability and the recovery were used. The MDL is defined by the U.S. Environmental Protection Agency as the minimum concentration of an analyte that can be reported greater than zero with a confidence of 99 % (U.S., 2009). Repeatability is shown at two concentration levels (0.1 ng g^−1^ and 10 ng g^−1^) as the relative standard deviation of a seven-fold measurement. All results are summarized in Table 1.

**Table 1:** Method Validation results.

Analytes	repeatability at 0.1 ng g^−1^ in %	repeatability at 10 ng g^−1^ in %	recovery at 10 ng g^−1^ in %	MDL/ng g^−1^
Dimethylsulfide	6	8	83	2.0
2-Butanol	3	6	97	2.5
Octane	10	9	86	0.8
Ethanol	7	9	94	2.2
Octanoic acid	9	6	86	2.0
Octanal	13	9	62	3.0
Linalooloxide	11	8	100	1.5
Benzaldehyde	4	4	110	4.6
Benzoic acid	9	9	86	3.4
Nonanol	9	9	96	4.7
2-Phenylethanol	11	9	100	2.7
Nonanoic acid	12	6	96	4.3
Thymol	12	7	98	1.6
Carvacrol	8	9	96	1.0

The repeatability at the lower concentration level showed already satisfying results with an average of 9 %, but with six analytes above 10 %. The results for the higher level showed an average of 8 % and no analyte above 10 % which shows great precision at this concentration level. The recovery was measured at 10 ng g^−1^ and showed good results for nearly all analytes. Only Octanal showed poor recovery with only 62 %. In average, the recovery was 92 %. The MDL varies a lot between the different analytes (0.8–4.7 ng g^−1^). Odeh an collaborators (Odeh, Abu-Lafi, & Al-Najjar, 2013) used a HS-SPME method for the identification of different honeys. They showed low limits of quantification (LODs, < 8.4 ng/g honey) by using the signal-to-noise ratio of three. As the calculations of the detection limits were done in two different ways, the comparison is difficult. Nevertheless, they are in the same order of magnitude. Four analytes can be compared with the ITEX-DHS method, namely octanoic acid, nonanoic acid, benzaldehyde and phenylethanol. When the detection limits of these four substances are compared, even though the comparison is difficult as the used methods to determine the LOD were different, it can be shown that for octanoic acid and nonanoic acid the presented ITEX-DHS method shows a smaller detection limit, but for benzaldehyde and phenylethanol the HS-SPME method shows the smaller LODs. The reported repeatability of HS-SPME was between 2 and 23 %. Only nonanoic acid shows a better reproducibility for HS-SPME than for ITEX-DHS. The ITEX-DHS method shows thus a better repeatability than the HS-SPME method.

### 3.2 Quantitative analysis

For quantification, all 14 analytes were investigated in the 32 honey samples with known botanical origin (acacia (A), blossom (B) and forest (F)). The range of the detected concentration ranges from nanogram per gram to microgram per gram. For each group of honey type, a mean was calculated. Table 2 summarizes the average concentrations of the different groups, the maximum concentration, the minimum concentration, the mean, the median and the sample number, in which the analyte was found.

**Table 2:** Lowest and highest detected analyte concentration, mean, median, number of samples in which the analytes have been found and average concentration of analytes in the three different honey varieties (acacia (A), blossom (B) and forest (F).

	lowest c ng g^−1^	highest c ng g^−1^	Mean ng g^−1^	Median ng g^−1^	samples (n)	ng g^−1^		
						Mean (A)	Mean (B)	Mean (F)
Dimethyl-sulfide	nd	nd	nd	nd	nd	nd	nd	nd
2-Butanol	135	2580	760	920	32	1450	845	860
Octane	0.9	1.6	1.1	1.1	32	1.3	1.1	1.2
Ethanol	3.2	690	50	160	6	nd	185	110
Octanoic acid	220	1255	500	635	11	590	835	530
Octanal	4.5	4.5	4.5	4.5	1	nd	4.5^+^	nd
Linalool-oxide	2.4	90	31	40	29	25	47	25
Benzaldehyde	7.5	120	28	34	32	47	32	30
Benzoic acid	12	450	120	130	28	38	150	150
Nonanol	6.0	3960	490	930	19	660	870	1370
2-Phenyl-ethanol	170	4515	1320	1535	32	1960	1450	1570
Nonanoic acid	24	1615	390	400	30	540	310	690
Thymol	1.8	4.8	2.2	2.8	6	2.7	2.8	nd
Carvacrol	1.1	5.6	2.0	2.7	8	nd	2.9	1.1^+^

*nd = not detected.

+only one sample was detected in this group and represents the mean.

Dimethylsulfide was found in none of the real samples. Ethanol was found in six different samples, but not in acacia honey. It is often identified in different honey types which leads to the idea, that it indicates fermentation processes and is not related to a specific honey type (Piasenzotto, Gracco and Conte 2003). 2-Butanol, octane, benzaldehyde and 2-phenylethanol were found in all 32 samples. Benzaldehyde, octane and 2-phenylethanol are known to be ubiquitous, so they were expected to be found in all samples (Machado & Miguel, 2020). Octanal was only found in one of the blossom honeys. For all other real samples, the detected concentrations were below the MDL. Some analytes do not show a big variation compared to the other botanical groups, as for example octane, linalooloxide, benzaldehyde, thymol and carvacrol. On the other hand, analytes like 2-phenylethanol, nonanol, octanoic acid and 2-butanol vary a lot between the groups and can thus help to find differences between honey varieties. The forest honey shows the highest concentrations in nonanol and nonanoic acid, which is typical for Greek forest honeys, whereas the acacia honey shows the highest concentration in 2-phenylethanol and 2-butanol, which are especially present in Spanish acacia honeys (Machado & Miguel, 2020). Octanoic acid was present in high concentrations in the blossom honeys. The comparison of the quantitative data to other publications is difficult, as often only qualitative analysis was done, and the chromatograms, which were achieved by different methods, were used for fingerprinting approaches (Radovic et al., 2001, Tomás-Barberán et al., 2001).

Ciotlaus et al. measured chromatographic profiles of volatiles of multifloral and unifloral honeys using HS-SPME. Validation results were not presented, so that the method sensitivity and repeatability cannot be compared. Furthermore, they did not quantify the identified components in the honeys and only presented their percentage amount (Ciotlaus, Balea, Pojar-Fenesan, & Petean, 2020). They found multiple components, including some of the analytes used in this study. Dimethyl sulfide was found in a small percentage of 1.8 % in multifloral honey, but not in unifloral honeys. Benzaldehyde, linalool oxide and nonanoic acid were found in both, multifloral and unifloral honey in similar percentages. This matches the result of this study, as all three analytes were found in all groups. Benzaldehyde and linalool oxide did not vary much in quantity, which matches the results of Ciotlaus et al., but nonanoic acid showed higher concentrations for forest honey samples in this study. Nonanol was only found in the multifloral honeys, which does not match the results of this study. Nonanol was also found in acacia honeys in this study, which is a monofloral honey. Octanoic acid was found in multifloral honey as well in acacia honey, in small quantities. Here, octanoic acid was found in all three studied honey groups but showed a higher concentration for the blossom honeys. Thymol was only present in a very small quantity in the multifloral honeys, sunflower honey and linden honey. The latter two honey types were not investigated in this study and thus, no comparison can be done for them. Anyhow, in this study, thymol was found in blossom honey in small concentration, which matches the result of the multifloral honeys. Additionally, it was detected in acacia honeys in very low concentration, which is a difference to Ciotlaus et al., who were not able to identify this compound for acacia honeys. This might be explained by the increased sensitivity of the ITEX-DHS method, but as no validation results were presented by Ciotlaus et al., this is only an assumption, and cannot be proved. The last compound which overlaps the two studies, is ethanol. It was detected in rape honey, which was not investigated with ITEX-DHS. However, both methods could not detect ethanol in acacia honey, which leads to the conclusion, that ethanol is not present in this honey type. Karabagias et al. studied different monofloral honeys, as well as a multifloral honey, using HS-SPME. They quantified based on an internal standard, so that the quantities are difficult to compare. Only four analytes overlap the studies, and for all, the concentration differ a lot. Benzaldehyde, Octanol and octane were found in higher concentrations (280, 240 and 1860 ng g^−1^, respectively) in the study of Karabagias et al., whereas nonanol showed a smaller concentration (70 ng g^−1^) (Karabagias, Badeka, & Kontominas, 2020). Ouradi et al. studied different types of honey from morocco, including different monofloral honeys as well as some mutlifloral honey, using HS-SPME. Ethanol was found in all monofloral honeys, but not in the mutlifloral ones. This does not match with the results found using ITEX-DHS, as in the blossom honeys, ethanol was detected. Ouradi et al. presents only four analytes per honey type, as these were found in the largest quantities. For the multifloral honeys, only linalool oxide overlaps with the analytes studied with ITEX-DHS. In the monofloral honey samples, benzoic acid, nonanoic acid and octanoic acid were detected, but no acacia honey was studied and thus the comparison to the here presented honey samples is not possible (Ouradi, Hanine, Fauconnier, Kenne, Rizki, Ennahli, & Hssaini, 2021).

**Fig. 2:**
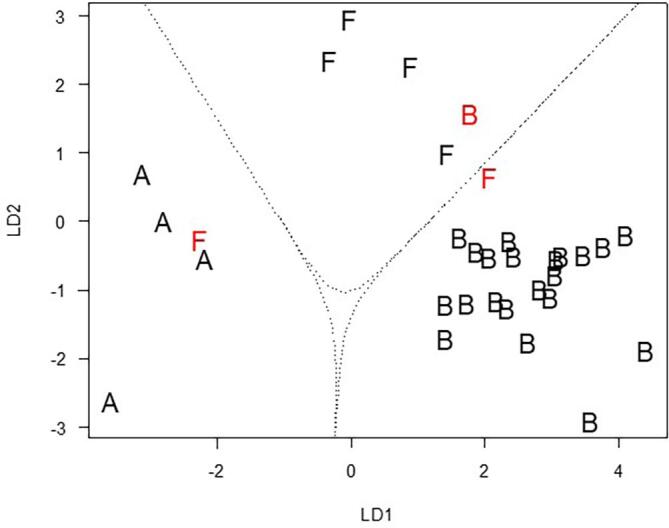
Scatterplot of the first two linear discriminant functions (LD) with the boundaries for each honey type. Falsely classified samples are shown in red. A: acacia honey, B: blossom honey, F: forest honey. (For interpretation of the references to color in this figure legend, the reader is referred to the web version of this article.)

The intention of the presented method was to create a quantitative method for the analysis of honey samples. Fig. 2 shows the overlaid chromatograms of three different honey samples (acacia, blossom and forest honey). It shows that with this method multiple peaks are detected, which were not identified in this study. The chromatograms of the different honeys show small differences, which could be used for fingerprinting approaches. Nevertheless, the focus of this study was the quantification of fourteen analytes. The statistical approach which was chosen for differentiation between honey types was the LDA with these 14 analytes.

### 3.3 Linear discriminant analysis

LDA is a commonly used chemometric tool to classify groups. The LDA performs a calculation, finding the smallest variance within a group, but the biggest variances between the different groups (McLachlan, 2004). The LDA is first performed with a training data set, where the groups are known. Afterwards, the test data can be loaded, and unknown samples can be identified.

To perform LDA, the free software R 3.6.2 (The R Foundation for Statistical Computing, https://www.r-project.org) was used, including the R-package “MASS” (Venables & Ripley, 2002). The peak areas of the 14 analytes were used as the explanatory variables for the LDA. The 38 honey samples were divided into four groups: acacia honey (A, 4), forest honey (F, 6), blossom honey (B, 22) and unknown honeys (U, 6). The last group wasn’t used for the training of the linear function, as there the honey type was not defined and should be discovered by applying the samples to the model.

The number of linear discriminant functions (LD) is limited to the number of groups minus one, leading to only two discriminant functions in the application. The LDs can be used for prediction resulting in a proportion of correct predictions of 90.6 %. The different loadings on the LDs are presented in Table 3. 2-Butanol was the main loadings on LD1 and LD2 into the negative direction. For LD1 the main loading into positive direction is given to dimethylsulfide and to nonanoic acid for LD2.

**Table 3:** Main loadings of LD1 and LD2.

Compound	LD1	LD2
Dimethylsulfide	1.60E + 06	8.117E-08
2-Butanol	−2.66E-06	−6.97E-06
Octane	2.61E-07	1.506E-07
Ethanol	2.737E-07	−1.08E-06
Octanoic acid	−1.54E-06	8.652E-07
Octanal	−2.23E-06	−1.31E-06
Linalooloxide	3.409E-07	−4.56E-07
Benzaldehyde	1.355E-07	−2.2E-08
Benzoic acid	2.351E-06	2.608E-07
Nonanol	2.983E-06	6.648E-07
2-Phenylethanol	−8.97E-07	6.728E-07
Nonanoic acid	1.243E-06	1.798E-06
Thymol	−1.45E-06	−1.37E-06
Carvacrol	1.76E-06	1.28E-07

Furthermore, the model can be used to show into which groups the unknown samples belong. The real groups and the predicted groups are presented in Table 4. 95.5 % of group B is correctly grouped, as well as 100 % of group A. Only in the forest honeys, the correct predictions are only 66.7 %, as two honeys are wrongly classified.

**Table 4:** Predicted and real varieties of the different samples.

Variety	Predicted variety			Correct predictions (%)
	A	B	F	
A (n = 4)	4			100.0
B (n = 22)		21	1	95.5
F (n = 6)	1	1	4	66.7
total				90.6

To get a visual understanding, a scatter plot is shown in Fig. 2, which is based on LD1 and LD2. The dotted lines show the boundaries between the different honey types, which means that the border, which classifies a honey to one or the other type, is strictly defined. The three misclassifications are shown in red.

**Fig. 3:**
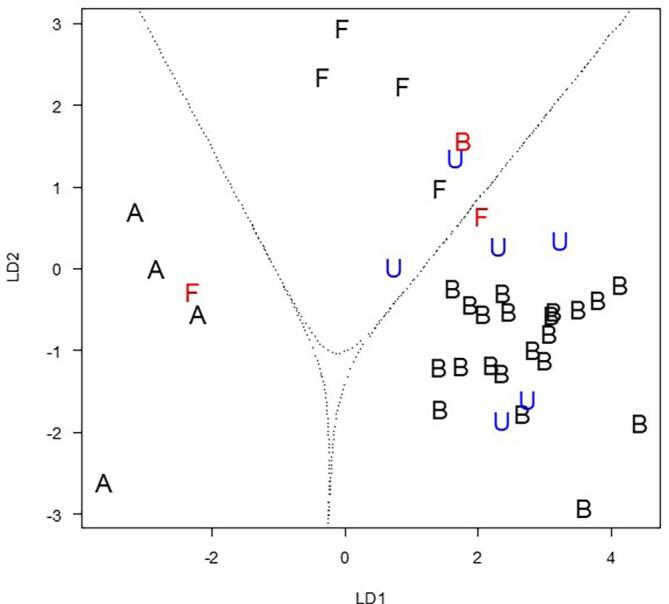
Scatterplot of the first two linear discriminant functions (LD) with the boundaries for each honey type. Falsely classified samples are shown in red. Unknown samples for prediction are shown in blue. A: acacia honey, B: blossom honey, W: forest honey, U: unknown honey.

As the model classifies groups with boundaries, unknown samples can be added into the graph, after performing LDA for these samples. As soon as they are added, it can be seen, to which group of honey they belong. This was done for six unknown honey samples, which are added in blue into Fig. 3. Two of the unknown honeys are predicted to be forest honeys and four of them to be blossom honey.

## 4 Conclusion and outlook

ITEX-DHS was successfully applied to the analysis of VOCs in honey. The reproducibility of the method was satisfying, as well as its recovery. ITEX-DHS is a very robust method, not only for the reproducibility results, but also in terms of mechanical stability and lifetime. The 38 measured honeys were used to create LDA for the classification into three honey types. Six unknown samples were classified using the prediction model. It would be of interest to increase the number of honey samples and honey types to ensure a more accurate model for the prediction by analyzing only a small number of analytes with a quick, robust and fully automated ITEX-DHS GC–MS method.

## Declaration of Competing Interest

The authors declare that they have no known competing financial interests or personal relationships that could have appeared to influence the work reported in this paper.
