# Supplementary material for: In-tube dynamic extraction for analysis of volatile organic compounds in honey samples
Source: Food Chem X. 2022 May 18;14:100337. doi: 10.1016/j.fochx.2022.100337 (PMC9130071; doi:10.1016/j.fochx.2022.100337)
Supplement: Supplementary Data 1 [file mmc1.docx]

**Supplemental material**

**In-tube dynamic extraction for analysis of volatile organic compounds in honey samples**

Wiebke Kaziur-Cegla^1^, Maik A. Jochmann^1^, Karl Molt^1^, Andreas Bruchmann^4^, Torsten C. Schmidt^1,2,3^

^1^Instrumental Analytical Chemistry, University of Duisburg-Essen, Universitätsstrasse 5, D-45141 Essen, Germany

^2^Centre for Water and Environmental Research (ZWU), University of Duisburg-Essen, Universitätsstr.2, 45141 Essen, Germany

^3^IWW Zentrum Wasser, Moritzstr. 26, 45476 Mülheim an der Ruhr, Germany

^4^Axel Semrau GmbH&Co.KG, Stefansbecke 42, 45549 Sprockhövel, Germany

Table S 1: Physicochemical properties of used compounds.

|  | *Cas no* | *purity* | *boiling point in* °C | *log k_wa_ 25°C* | *satchenow constant k_s_* | *quant. Ion m/z* | *qual. Ions m/z* |
| --- | --- | --- | --- | --- | --- | --- | --- |
| alcohols |  |  |  |  |  |  |  |
| Ethanol | 64-17-  5 | 98 | 78.32 | 3.54 | 0.12 | 45 | 46 |
| 2-Butanol | 78-9-2 | >99 | 99 | 3.35 | 0.17 | 45 | 59, 74 |
| Nonanol | 143-08-8 | 98 | 214 | 2.77 | 0.29 | 55 | 56 |
| Linalool | 60047-17-8 | 97 | 198 | 2.95 | 0.29 | 59 | 54, 11, 132 |
| Phenylethanol | 60-12-8 | 99 | 220 | 5.02 | 0.19 | 91 | 92, 122 |
| adehydes |  |  |  |  |  |  |  |
| Benzaldehyde | 100-52-7 | >99 | 179 | 3.12 | 0.18 | 77 | 105, 106 |
| Octanal | 124-13-0 | 99 | 171 | 1.85 | 0.27 | 55 | 56, 84 |
| alkanes |  |  |  |  |  |  |  |
| Octane | 111-65-9 | 99 | 126 | -1.98 | 0.32 | 43 | 57, 85, 114 |
| acids |  |  |  |  |  |  |  |
| Benzoic acid | 65-85-0 | 99.5 | 250 | 5.25 | 0.17 | 77 | 105, 122 |
| Octanoic acid | 124-07-2 | 96 | 237 | 4.13 | 0.25 | 43 | 55, 73 |
| Nonanoic acid | 112-05-0 | 99 | 255 | 4.01 | 0.27 | 57 | 60, 73 |
|  |  |  |  |  |  |  |  |
| Thymol | 89-83-8 | 99 | 232 | 4.27 | 0.24 | 91 | 135, 150 |
| Carvacrol | 499-75-2 | 98 | 237 | 4.64 | 0.24 | 91 | 135, 150 |
| Dimethylsulfide | 75-18-3 | 98 | 37 | 1.18 | 0.16 | 47 | 61, 62 |

*Table S2 Compilation of information of the included honeys.*

|  | **Name** | **Provider** | **Honey type** | **Origin** |  | **Expiry Date** |
| --- | --- | --- | --- | --- | --- | --- |
| A1 | REWE Bio Akazienhonig | Atrium Import GmbH | acacia honey | Mix from honey from EU-countries | e | 01.12.2019 |
| A2 | Bio Akazienhonig | Atrium Import GmbH | acacia honey | Mix from honey from EU-countries and non-EU-Countries | m | 26.02.2020 |
| A3 | Langnese Honig Akazie | Langnese Honig | acacia honey | Mix from honey from EU-countries | e | 01.11.2019 |
| A4 | Biophar Akazienhonig | Fürsten-Reform | acacia honey | Mix from honey from EU-countries and non-EU-Countries | m | Dez 19 |
| B01 | Blütenhonig flüssig | W.L. Ahrens GmbH & Co | blossom honey | Mix from honey from EU-countries and non-EU-Countries | m | 30.06.2018 |
| B02 | Lune de Miel Cremiger Blütenhonig | Famille Michaud Apiculteurs | blossom honey | Mix from honey from EU-countries and non-EU-Countries | m | 14.09.2019 |
| B03 | Vom Land Blütenhonig cremig | Vom Land für Netto Marken-Discount | blossom honey | Mix from honey from EU-countries and non-EU-Countries | m | 10.01.2019 |
| B04 | Glüsener Speerlese | Hans-Jürgen Speer | blossom honey | Tostedt | g |  |
| B05 | Bienenwirtschaft Meissen Bienen Gold Blütenhonig | Bienenwirtschaft | blossom honey | Mix from honey from EU-countries and non-EU-Countries | m | 01.01.2020 |
| B06 | heldere Bloemenhoning | Albert Heijn B.V | blossom honey | Mix from honey from EU-countries and non-EU-Countries | m | 12.01.2020 |
| B07 | Bloemen Honing | Ahold Delhaize | blossom honey | Mix from honey from EU-countries and non-EU-Countries | m | 21.03.2020 |
| B08 | Sophies | Sophie Simon | blossom honey | Rheinland-Pfalz, Bausendorf | g |  |
| B09 | Fair Blütenhonig cremig | Honigland GmbH | blossom honey | Mix from honey from non- EU-countries | a | Sep 19 |
| B10 | Langnese Honig Bergblüte | Langnese Honig | blossom honey | Mix from honey from EU-countries and non-EU-Countries | m | 01.11.2019 |
| B11 | REWE Bio Vielblütenhonig | Atrium Import GmbH | blossom honey | Mix from honey from EU-countries and non-EU-Countries | m | 24.10.2019 |
| B12 | Biophar Wabenquell Honig | Fürsten-Reform | blossom honey | Mix from honey from non- EU-countries | a | Okt 19 |
| B13 | Biophar Imkerhonig streichzart | Fürsten-Reform | blossom honey | Mix from honey from EU-countries and non-EU-Countries | m | Nov 19 |
| B14 | Biophar Honig aus Nord-Deutschland | Fürsten-Reform | blossom honey | Niedersachsen, Schleswig-Holstein | g | Aug 19 |
| B15 | Langnese Honig Sommerblüte | Langnese Honig | blossom honey | Mix from honey from EU-countries and non-EU-Countries | m | 01.11.2019 |
| B16 | Flotte Biene Wildblütenhonig | Langnese Honig | blossom honey | Mix from honey from EU-countries and non-EU-Countries | m | Jan 20 |
| B17 | Biophar Sanddorn in Blütenhonig | Fürsten-Reform | blossom honey | - | g | Aug 19 |
| B18 | Marlene Blütenhonig goldklar | Eystruper Land GmbH | blossom honey | Mix from honey from EU-countries and non-EU-Countries | m | 07.02.2019 |
| B19 | Zoran's Essener Blütenhonig | Berufsimkerei Zoran Krizic | blossom honey | Essen, Germany | g |  |
| B20 | Wildblumenhonig Mielbio | Mielbio Rigoni di Asiago S.r.l. | blossom honey | Asiago, Italy | i | 18.04.2021 |
| B21 | Kretischer Honig von Wildblüten, Koniferen und Thymian | Kreta Food GmbH & Co | blossom honey | Greece | e | 31.07.2019 |
| L1 | Biophar Lindenhonig | Fürsten-Reform | linden honey | Mix from honey from EU-countries and non-EU-Countries | m | 01.12.2019 |
| O1 | Orangenhonig Mielbio | Mielbio Rigoni di Asiago S.r.l. | orange honey | Asiago, Italy | i | 18.04.2021 |
| D1 | Dublin Town & Country | Lamberts Honey | Dublin Town & Country honey | Dublin, Ireland | e | - |
| U1 | GutBio Honig cremig | Honigland GmbH | unknown | Mix from honey from EU-countries and non-EU-Countries | m | Jun 19 |
| U2 | Lune de Miel Milder Honig (Bärenflasche) | Famille Michaud Apiculteurs | unknown | Mix from honey from EU-countries and non-EU-Countries | m | 15.11.2019 |
| U3 | Kristinas Honig 3 (Papa) | Unbekannt | unknown | unknown | g |  |
| U4 | GutBio Honig cremig (flüssig) | Honigland GmbH | unknown | Mix from honey from EU-countries and non-EU-Countries | m |  |
| W1 | Marlene Waldhonig feinwürzig | Eystruper Land GmbH | forest honey | Mix from honey from EU-countries and non-EU-Countries | m | 09.01.2019 |
| W2 | Waldhonig | Honigland GmbH | forest honey | Mix from honey from EU-countries and non-EU-Countries | m | Nov 18 |
| W3 | Kristinas Honig 1 (Holtke) | Dieter Holtke | forest honey | NRW, Westerkappeln | g |  |
| W4 | Melvita biologische Honing | Melvita honing | forest honey | Europa, Middle-Southamerica and Asia | m | 28.02.2020 |
| W5 | Waldhonig Mielbio | Mielbio Rigoni di Asiago S.r.l. | forest honey | Asiago, Italy | i | 18.04.2021 |
| W6 | Biophar Waldhonig | Fürsten-Reform | forest honey | Mix from honey from EU-countries and non-EU-Countries | m | Jan 20 |

| e | europe |
| --- | --- |
| m | mix all |
| g | germany |
| i | italy |
| a | asia |
